# GSK3β Is Involved in JNK2-Mediated β-Catenin Inhibition

**DOI:** 10.1371/journal.pone.0006640

**Published:** 2009-08-13

**Authors:** Dong Hu, Xiuli Bi, Wenfeng Fang, Anjia Han, Wancai Yang

**Affiliations:** 1 Department of Pathology, University of Illinois at Chicago, Chicago, Illinois, United States of America; 2 Department of Pathology, the First Affiliated Hospital and Zhongshan School of Medicine, Sun Yat-Sen University, Guangzhou, China; University of Texas MD Anderson Cancer Center, United States of America.

## Abstract

**Background:**

We have recently reported that mitogen-activated protein kinase (MAPK) JNK1 downregulates β-catenin signaling and plays a critical role in regulating intestinal homeostasis and in suppressing tumor formation. This study was designed to determine whether JNK2, another MAPK, has similar and/or different functions in the regulation of β-catenin signaling.

**Methodology and Principal Findings:**

We used an *in vitro* system with manipulation of JNK2 and β-catenin expression and found that activated JNK2 increased GSK3β activity and inhibited β-catenin expression and transcriptional activity. However, JNK2-mediated downregulation of β-catenin was blocked by the proteasome inhibitor MG132 and GSK3β inhibitor lithium chloride. Moreover, targeted mutations at GSK3β phosphorylation sites (Ser33 and Ser37) of β-catenin abrogated JNK2-mediated suppression of β-catenin. *In vivo* studies further revealed that JNK2 deficiency led to upregulation of β-catenin and increase of GSK3-β phosphorylation in JNK2-/- mouse intestinal epithelial cells. Additionally, physical interaction and co-localization among JNK2, β-catenin and GSK3β were observed by immunoprecipitation, mammalian two-hybridization assay and confocal microscopy, respectively.

**Conclusion and Significance:**

In general, our data suggested that JNK2, like JNK1, interacts with and suppresses β-catenin signaling *in vitro* and *in vivo*, in which GSK3β plays a key role, although previous studies have shown distinct functions of JNK1 and JNK2. Our study also provides a novel insight into the crosstalk between Wnt/β-catenin and MAPK JNKs signaling.

## Introduction

The Wnt/β-catenin signaling plays a critical role in embryonic development and the regulation of homeostatic self-renewal in a number of adult tissues [1]. However, an aberrant activation of Wnt/β-catenin signaling, such as β-catenin accumulation and nuclear translocation, leads to transactivation of transcription of a broad range of genes and contributes to carcinogenesis, although this signaling pathway could be regulated by other signaling, molecules or pathways, including a destruction complex consisting of casein kinase Iα (CKIα), glycogen synthase kinase 3β (GSK3β), adenomatous polyposis coli (APC) and Axin. We recently reported that mitogen-activated protein kinase (MAPK) JNK1 interacts with and negatively regulates β-catenin signaling through GSK3β [2], and that the β-catenin alteration is likely to be associated with intestinal tumor formation induced by JNK1 inactivation [2].

Recently, an increasing body of evidence supported the crosstalk between MAPK and Wnt/β-catenin signaling [3]–[8]. It was reported that p38 MAPK regulates canonical Wnt/β-catenin signaling by inactivation of GSK3β [7], [8]. However, JNK1, another important member of MAPK, was suggested to antagonize Wnt/β-catenin signaling by activating GSK3β [2].

The JNKs are encoded by three genes, namely *JNK1*, *JNK2*, and *JNK3*. JNK1 and JNK2 are ubiquitously expressed, whereas the expression of JNK3 is largely restricted to brain, heart, and testis [9]. Numerous studies have reported distinct functions of JNK1 and JNK2, for example, mice harboring JNK1 or JNK2 inactivation exhibited opposite susceptibility to tumor formation induced by 12-O-tetradecanoylphorbol-13-acetate (TPA) [10], [11]. In this study, we found that, like JNK1, JNK2 interacts with and inhibits β-catenin signaling, in which GSK3β functions upstream of proteasome pathway and plays a critical role in JNK2-mediate β-catenin degradation.

## Materials and Methods

### Cell Culture, Plasmids Construction and Transfection

Human embryonic kidney (HEK) 293T cells were maintained in Dulbecco's modified Eagle's medium, human lung cancer cells A549 were grown in minimal essential medium. pcDNA3-Flag-MKK7-JNK2 (active JNK2) and pcDNA3-Flag-MKK7-JNK1 (active JNK1) were provided by Dr.Davis [12], pcDNA3-hemagglutinin (HA)-β-catenin, HA-tagged β-catenin mutants (HA-S33Y β-catenin, HA-S33F β-catenin and HA-S37A β-catenin), pACT-JNK2 and pBIND- β-catenin were constructed as described previously [2], [13]–[15]. TCF-4 reporter plasmid TOPFLASH and the control inactive reporter FOPFLASH were purchased from Upstate Biotechnology (Lake Placid, NY). Reporter plasmid pTK-Renilla was purchased from Promega (Madison, WI). For transfection, cells were plated to form a 50–70% confluent culture. The HEK293T cells were transfected using Lipofectamine 2000 (Invitrogen Corp.). For the A549 cells, transfection was carried out by Lipofectamine combined with Plus reagent (Invitrogen Corp.).

### Immunoblotting

Standard immunoblotting analysis was used as described [2]. Blots were probed with the antibodies specific for HA, p-JNK, p-c-Jun, β-catenin (Santa Cruz Biotechnology), GSK3β, p-GSK3β (Cell Signaling Technology, Beverly, MA) and β-actin (Sigma–Aldrich Corp.) and the bands were visualized using enhanced chemiluminescence plus reagents (Amersham Pharmacia, Piscataway, NJ).

### TCF-4 Reporter Assay

Cells were co-transfected with pcDNA3-HA-β-catenin and pcDNA3-Flag-MKK7-JNK1 or pcDNA3-Flag-MKK7-JNK2, along with TOPFLASH or FOPFLASH. 24 or 48 h after transfection, the cells were harvested to conduct a firefly and *Renilla* luciferase activity assay using a dual luciferase kit (Promega). The TCF-4 reporter activity was presented as by ratio of firefly to *Renilla* luciferase activity. Each experiment was triplicated independently.

### Mouse Intestinal Epithelial Cell Isolation and Immunoblotting

Similar as we described previously [16], intestinal epithelial cells were isolated from JNK2+/+ and JNK2-/- mice [17] small intestine by incubating with 15 mM EDTA buffer. Resulting cell pellets were lysed for immunoblotting for JNK2, β-catenin, GSK3β, p-GSK3β, CDK4 (Santa Cruz Biotechnology, Santa Cruz, CA) and β-actin analysis.

### Immunoprecipitation

As described [16], whole-cell lysates were incubated with specific anti-Flag antibody to pull down MKK7-JNK2 using the Catch and Release reversible Immunoprecipitation system (Upstate Biotechnology, Lake Placid, NY), followed by immunoblotting probed with anti-HA (for β-catenin) or anti-GSK3β (for GSK3β).

### Mammalian two-Hybridization Assay

HEK293T cells were plated at a density of 2.5×10^5^ cells/well in 24-well plate the day before transfection. Two µg of pACT-JNK2, pBind-β-catenin and pG5luc were co-transfected using Lipofectamine 2000 (Invitrogen, CA). pACT and pBind were used as negative controls. To eliminate non-specific interactions, two groups of negative controls were set: pACT-JNK2 and pBind, pACT and pBind-β-catenin. After 48 h, samples were lysed using 1x Passive lysis buffer, and the amount of firefly luciferase and Renilla luciferase was quantified using the Dual-Luciferase Reporter Assay System (Promega Corporation, Masison, WI), as we described recently [15]. The experiments were triplicated independently.

### Immunofluorescence Staining

As described recently [2], HEK293T cells were co-transfected with pEGFP-β-catenin and pcDNA3-Flag-MKK7-JNK2. Twenty-four h after transfection, cells were fixed and stained with anti-Flag antibody (1⊗200; Genescript, Piscataway, NJ), followed by incubating with tetramethyl rhodamine isothiocyanate (TRITC) labeled anti-mouse IgG (Santa Cruz Biotechnology), mounted with UltraCruz 4′-6-Diamidino-2-phenylindole (DAPI) containing mounting medium (Santa Cruz Biotechnology), visualized under a Zeiss LSM510 META confocal microscope (Zeiss, Jena, Germany). Images were taken and analyzed using Zeiss LSM Image Browser.

## Results and Discussion

### Activated JNK2 Suppressed Wnt/β-catenin Expression and Transcriptional Acticcity

**Figure 1:**
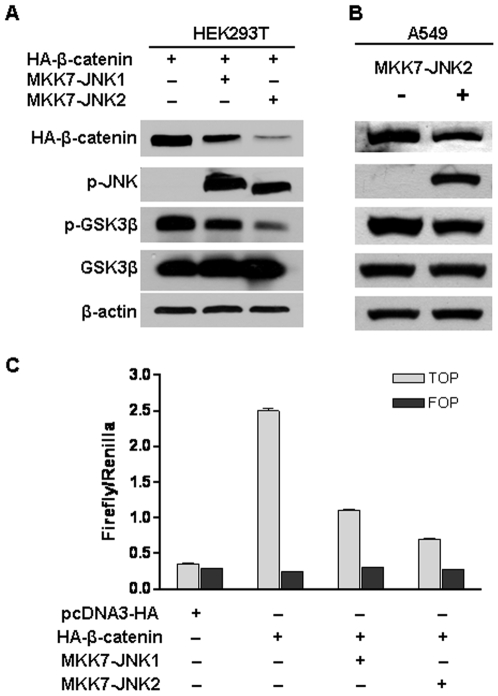
Active JNK2 downregulated β-catenin expression, inhibited its transcriptional activity and reduced GSK3β phosphorylation. (A) Active JNK2 suppressed β-catenin expression and GSK3β phosphorylation in HEK293T cells. HEK293T cells were transfected with pcDNA3-HA-β-catenin together with pcDNA3-Flag-MKK7-JNK1 or pcDNA3-Flag-MKK7-JNK2. Forty-eight hours after transfection, cells were harvested for immunoblotting analysis to detect the alterations of HA-β-catenin, p-JNK, p-c-Jun, phospho-Ser^9^ GSK3β, and GSK3β. β-actin served as loading control. (B) Active JNK2 reduced GSK3β phosphorylation and downregulated β-catenin expression in human lung cancer cell line A549. A549 cells were co-transfected with pcDNA3-HA-β-catenin and pcDNA3-Flag-MKK7-JNK2. Forty-eight hours after transfection, cells were harvested for immunoblotting analysis to detect the alterations of β-catenin, p-JNK, and phospho-Ser^9^ GSK3β. β-actin served as loading control. (C) Active JNK inhibited β-catenin-mediated transcriptional activity of TCF. HEK293T cells were co-transfected with pcDNA3-Flag-MKK7-JNK1 or pcDNA3-Flag-MKK7-JNK2, pcDNA3-HA-β-catenin, TOPFLASH (TOP) or FOPFLASH (FOP), and Renilla. 48 h after transfection, cells were harvested for luciferase activity assay. Each bar represents the mean ± standard deviation (SD) for triplicated samples.

The studies from us and others have demonstrated that JNK1 can antagonize the canonical Wnt/β-catenin signaling [2], [4]. To elucidate the potential role of JNK2 in the regulation of Wnt/β-catenin signaling, constitutively active JNK2 (MKK7-JNK2) was co-transfected with β-catenin into HEK293T cells. As shown in figure 1A (Lane 3 versus lane 1), β-catenin protein level was dramatically reduced in MKK7-JNK2-transfected HEK293T cells, even to a greater extent than that in MKK7-JNK1-transfected cells (Figure 1A, lane 3 versus 2), suggesting that both JNK1 and JNK2 activation downregulate β-catenin expression although to a different extend.

To determine whether activated JNK2 can inhibit the aberrantly accumulated nuclear β-catenin in cancer cells, a human lung cancer cell line A549 was transfected with MKK7-JNK2. Immunoblotting analysis showed a reduction of endogenous β-catenin protein (Figure 1B), which is consistent with the observation made in HEK293T cells. However, the degree of β-catenin suppression in A549 was less than that in HEK293T cell, which might be a result of biological differences between HEK293T cells and the transformed human lung cancer cells.

β-catenin interacts with TCF-4 and stimulates gene expression. To determine whether JNK2 activation can affect the β-catenin-driven gene transription, MKK7-JNK2 was co-transfected with β-catenin into HEK293T cells along with TCF-4 reporter plasmid TOPFLASH or the inactive control FOPFLASH. TOPFLASH includes a luciferase reporter driven by three copies of TCF binding elements upstream of the thymidine kinase (TK) minimal promoter, and is specifically regulated by Wnt/β-catenin signaling [18]. TOPFLASH used here is different from LEF1-Luciferase reporter used in a recent report [6]. The latter contains seven LEF1 binding sites, in p301fosCAT plasmid, containing the minimal fos promoter linked to the chloramphenicol accetyltransferase (CAT) gene [19]. Upon transfection of β-catenin, the TOPFLASH reporter activity increased by about 10-fold (Figure 1C), although no change was observed in the cells transfected with the empty pcDNA3-HA control vector. However, MKK7-JNK2 transfection strongly suppressed the β-catenin-induced TOPFLASH reporter activity even to a greater extent than MKK7-JNK1 (Figure 1C). The similarities between the regulations of β-catenin-driven transactivation and β-catenin protein level by JNK1 and JNK2 activation implicates that, the inhibitory effect of activated JNK2 on β-catenin-driven transactivation is probably attributed to the downregulation of β-catenin protein. Taken together, these data demonstrate for the first time a negative regulation of Wnt/β-catenin signaling by JNK2 activation.

**Figure 2:**
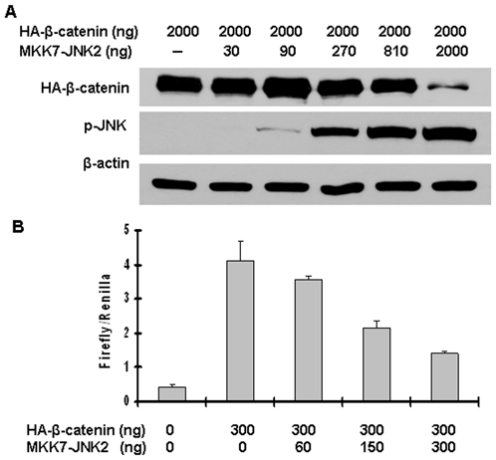
Active JNK2 downregulated β-catenin expression and inhibited its transcriptional activity in a dose-dependent manner. (A) Activated JNK2 reduced β-catenin protein level in a dose-dependent manner. HEK293T cells were co-transfected with pcDNA3-HA-β-catenin along with different amounts of pcDNA3-Flag-MKK7-JNK2, as indicated. Forty-eight hours after transfection, cells were harvested for immunoblotting analysis to detect the alterations of HA-β-catenin and p-JNK. β-actin served as loading control. (B) Activated JNK2 inhibited β-catenin-mediated transcriptional activity of TCF in a dose-dependent manner. HEK293T cells were co-transfected with pcDNA3-HA-β-catenin, TOPFLASH, Renilla, along with different amounts of pcDNA3-Flag-MKK7-JNK2, as indicated. Forty-eight hours after transfection, cells were harvested for luciferase activity assay. Each bar represents the mean ± standard deviation (SD) for triplicated samples.

To confirm the suppression of Wnt/β-catenin signaling by JNK2 activation, a constant level of β-catenin was co-transfected with increasing amounts of MKK7-JNK2 into HEK293T cells. The alterations of exogenous β-catenin were assessed by immunoblotting. As shown in figure 2A, a slight decrease in β-catenin protein level was seen with 810 ng of pMKK7-JNK2, and a dramatic reduction in β-catenin protein level was observed with higher amounts of pMKK7-JNK2. The effect of increasing amounts of MKK7-JNK2 on β-catenin-induced transaction was also evaluated using a luciferase activity assay. As shown in figure 2B, co-transfection of 150 ng of pMKK7-JNK2 suppressed the TOPFLASH reporter activity by 50% and 300 ng suppressed it by 65%. Taken together, these data demonstrate a dose-dependent inhibition of Wnt/β-catenin signaling by JNK2 activation.

### Activated JNK2 Reduces β-catenin Protein Level Through Proteasomal Degradation

**Figure 3:**
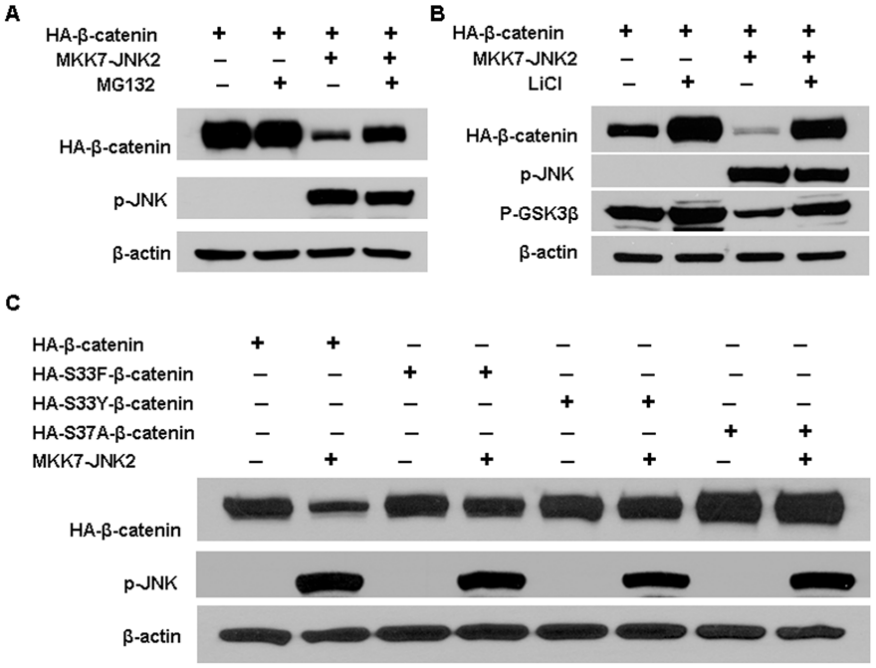
Active JNK2-mediated β-catenin degradation occurred through the proteasome system and GSK3β. (A) HEK293T cells were co-transfected with pcDNA3-HA-β-catenin and pcDNA3-Flag-MKK7-JNK2 (lane 3 and 4) or empty vector (lane 1 and 2). Forty-four hours after transfection, 25 µM MG132 was added to the indicated samples (lane 2 and 4). Four hours later cells were harvested for immunoblotting analysis to detect the expression of HA-β-catenin and p-JNK. (B) Blocking GSK3β activity by LiCl reduced β-catenin expression inhibition by activated JNK2. pcDNA3-HA-β-catenin was transfected into HEK293T cells along with pcDNA3-Flag-MKK7-JNK2 (lane 3 and 4) or empty vector (lane 1 and 2). Thirty-six hours after transfection, half of the cultures were treated overnight with 30 mM LiCl (lane 2 and 4) and then harvested for immunoblotting analysis to detect the expression of HA-β-catenin, phospho-Ser-9 GSK3β, and p-JNK. (C) Mutant β-catenin was resistant to activated JNK2 induced degradation. Wild-type β-catenin (HA- β-catenin) (lanes 1 and 2) or various β-catenin mutants (HA-S33F β-catenin, lanes 3 and 4; HA-S33Y β-catenin, lanes 5 and 6; HA-S37A β-catenin, lanes 7 and 8) were transfected into HEK293T cells along with pcDNA3-Flag-MKK7-JNK2 (lane 2,4,6,8) or empty vector (lanes 1,3,5,7). 48 hours after transfection, cells were harvested for immunoblotting analysis to determine the protein levels of HA-β-catenin. β-actin served as loading control.

Cytoplasmic β-catenin protein levels are regulated by ubiquitination and subsequent proteasomal degradation. To determine whether the decrease of β-catenin protein induced by JNK2 activation is due to increased proteasomal degradation, a proteasomal inhibitor MG132 [20] was used to treat HEK293T cells after transfection with MKK7-JNK2 and β-catenin. As shown in figure 3A, MG132 blocked JNK2-induced β-catenin inhibition (lane 4 versus 3), demonstrating that JNK2-caused β-catenin degradation was through proteasomal degradation system.

### GSK3β Functions Upstream of the Proteasome to Mediate the Reduction of β-catenin by JNK2 Activation

Multiple pathways have been reported to mediate β-catenin degradation by the proteasome, such as p53/Siah-1/APC, Wnt/GSK3β/APC, and retinoid X receptor (RXR)-mediated pathway [21]. To determine the role of the most common GSK3β-regualated pathway in the downregulation of β-catenin by JNK2 activation, we detected the phosphorylation status of GSK3β. GSK3β could be inactivated by phosphorylation at Ser-9 by serine/threonine kinases, such as Akt, protein kinase A (PKA), and protein kinase C (PKC), followed by decreased activities [22]–[24]. Thus, GSK3β phosphorylation at Ser-9 has been used as an indirect marker of decreased GSK3β activity. As shown in figure 1A, Ser-9 phosphorylation of GSK3β (p-GSK3β) was indeed decreased in MKK7-JNK2-transfected HKE293T cells, which meant that GSK3β activity was increased by activated JNK2. The expression pattern of p-GSK3β is similar to that of β-catenin, leading us to postulate that the β-catenin degradation is attributed to the increased GSK3β activity induced by JNK2 activation. In fact, increased GSK3β activity and corresponding downregulation of β-catenin were also seen in A549 cells (Figure 1B).

To further test our hypothesis, GSK3β inhibitor lithium chloride (LiCl) was used to suppress GSK3β activity and the effect of activated JNK2 on β-catenin expression was analyzed. As shown in figure 3B, LiCl inhibited GSK3β activity which was indicated by an increase of GSK3β phosphorylation (lane 2 versus 1; lane 4 versus 3). The ability of activated JNK2 to downregulate β-catenin was almost completely abrogated upon LiCl treatment (lane 4 versus 3), suggesting that GSK3β is required for JNK2-mediated β-catenin degradation.

Post-translational modifications play critical role in the regulation of the β-catenin turnover by GSK3β [25]. To further confirm the role of GSK3β in β-catenin degradation induced by JNK2 activation, we generated series of constructs expressing β-catenin mutants [2] and co-transfected them with pMKK7-JNK2 into HKE293T cells. As shown in figure 3C, S33Y, S33F, or S37A mutation endowed β-catenin with less susceptibility to degradation induced by JNK2 activation, further demonstrating the requirement of GS3β for JNK2-mediated degradation of β-catenin.

Besides the indirect phosphorylation and degradation of β-catenin by JNK2 via GSK3β from our present study, a recent study has identified three more phosphorylation sites in β-catenin protein, two of them (Ser-191 and Ser-605) are critical for β-catenin stabilization and nuclear localization, and could be directly phosphorylated by JNK2, providing more evidences of the different roles of JNK2 in Wnt/β-catenin signaling [6]. However, JNK1 is not likely to have such a strong influence to phosphorylate Ser-191/605 of β-catenin [6].

### Genetic Deficiency of JNK2 caused Upregulation of β-catenin, CDK4 and GSK3β Phosphorylation

**Figure 4:**
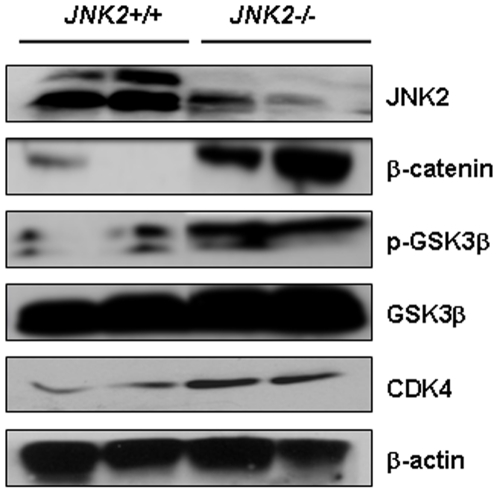
JNK2 deficiency caused upregulation of β-catenin and its downstream target CDK4, as well as upregulation of GSK3β phosphorylation in JNK2-/- mouse intestinal epithelial cells, compared to those in JNK2+/+ mice. Each lane represents one mouse. β-actin served as loading control.

To validate the regulation β-catenin by JNK2 *in vivo*, JNK2-/- and +/+ mice were dissected. As shown in figure 4, β-catenin and its downstream target CDK4 were upregulated in intestinal epithelial cells from JNK2-/- mice, compared to the JNK2+/+ mice. Consistently, phosphorylated GSK3β was upregulated although total GSK3β was not changed. The *in vivo* data provided additional evidence that GSK3β is involved in JNK2-mediated β-catenin regulation.

As to the effect of JNK2 on β-catenin downstream targets, our most recent *in vitro* study demonstrated that selenium-stimulated phosphorylation of JNK1/2 dramatically inhibited β-catenin and its downstream targets, such as c-myc, cyclin D1 and CDK4, in human colon cancer cells (Fang and Yang, et al, submitted), supporting the notion of activated JNK1 and JNK2 in the suppression of β-catenin signaling.

### Active JNK2 Directly Interacts with GSK3β and β-catenin

**Figure 5:**
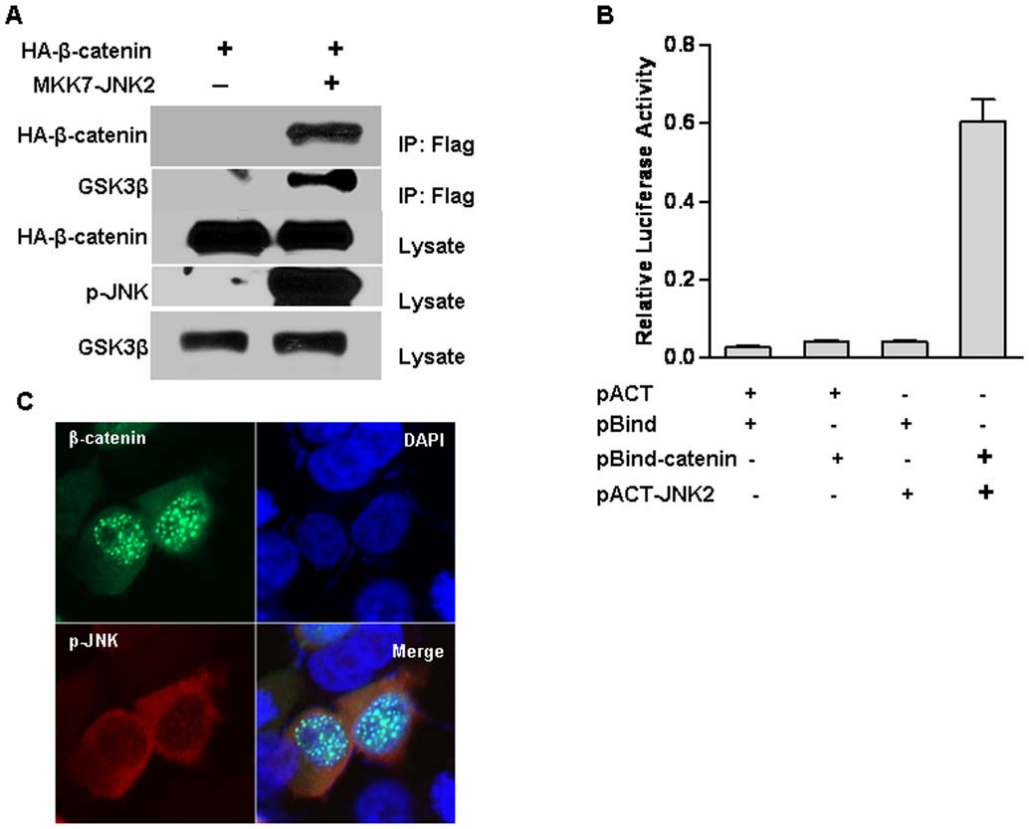
Activated JNK2 interacts with β-catenin and GSK3β. (A) Active JNK2 binding to β-catenin and GSK3β was analyzed by immunoprecipitation. β-catenin (HA tagged) was co-transfected with empty vector or active JNK2 (Flag tagged) into HEK293T cells. Immunoprecipitation was performed with a Flag antibody. (B) Mammalian two-hybridization assays showed a strong binding of β-catenin and JNK2 protein. The experiments were triplicated independently. (C) Active JNK2 and β-catenin co-localized in the cell nucleus and cytoplasm. Active JNK2 (Flag tagged) and pEGFP-β-catenin were co-transfected into HEK293T cells. The cells were immunostained with a Flag antibody. Co-localization (yellow fluorescence) of active JNK2 (red fluorescence) and β-catenin (green fluorescence) was detected in the nucleus and cytoplasm.

Although our data strongly demonstrate that JNK2 suppresses β-catenin signaling through GSK3β-proteasome pathway, the mechanism underlying the regulation of GSK3β activity by active JNK2 remains elusive. In addition, we can not exclude the possibility that JNK2 can directly interact with β-catenin. Therefore, immunoprecipitation assays were carried out to test a potential interaction between JNK2, GSK3β, and/or β-catenin. As shown in figure 5A, both β-catenin and GSK3β can be seen in the precipitate by anti-Flag tag antibody (lane 2), but they were absent in the cells without Flag-tagged constitutively active JNK2 (lane 1), indicating that active JNK2 indeed interacted with GSK3β and β-catenin. It is possible that JNK2, GSK3β and β-catenin form a multi-protein complex since cytoplasmic interaction of GSK3β and β-catenin has been found [26], [27]. The interaction between JNK2 and β-catenin was further validated by mammalian two-hybridization system (Figure 5B) and co-localization analysis (Figure 5C), as we reported recently [2]. As shown in figure 5C, active JNK2 was co-localized with β-catenin in the nucleus and cytoplasm, consistent with the results from the immunoprecipitation assays.

Since overexpressed β-catenin is mainly restricted in the nucleus as shown in figure 5C and reported previously [2], [4], it is postulated that activated JNK2 promotes β-catenin degradation by increasing GSK3β and proteasome activity and potentially degrading β-catenin in the cytoplasm by driving β-catenin translocation. In fact, JNK1 was also reported to antagonize Wnt/β-catenin signaling by expelling β-catenin out of nucleus [4] and promoting its degradation [2]. However, a recent report revealed that JNK2 interacts with β-catenin and mediates the phosphorylation of β-catenin by Rac1, which is critical for the nuclear accumulation of β-catenin in response to Wnt signaling [6]. Comparing the present study to our recent findings and other studies, we postulate that the role of JNK1 or JNK2 in canonical Wnt signaling depends on the duration, degree, tissue, and/or subcellular location of its activation.

In conclusion, our present data demonstrate that activated JNK2 promoted β-catenin degradation and inhibited the canonical Wnt/β-catenin signaling *in vitro*, and that JNK2 deficiency upregulated β-catenin signaling *in vivo*, in which GSK3β is likely to play a critical role. Our study also provides a novel insight into the crosstalk between Wnt/β-catenin and MAPK JNKs signaling.
